# Genome Sequence and Annotation of *Colletotrichum higginsianum*, a Causal Agent of Crucifer Anthracnose Disease

**DOI:** 10.1128/genomeA.00821-16

**Published:** 2016-08-18

**Authors:** Antonios Zampounis, Sandrine Pigné, Jean-Félix Dallery, Alexander H. J. Wittenberg, Shiguo Zhou, David C. Schwartz, Michael R. Thon, Richard J. O’Connell

**Affiliations:** a UMR BIOGER, INRA, AgroParisTech, Université Paris-Saclay, Thiverval-Grignon, France; b KeyGene N.V., Wageningen, The Netherlands; c Laboratory for Molecular and Computational Genomics, Department of Chemistry, Laboratory of Genetics, University of Wisconsin–Madison, Madison, Wisconsin, USA; d Instituto Hispano-Luso de Investigaciones Agrarias (CIALE), Department of Microbiology and Genetics, University of Salamanca, Salamanca, Spain

## Abstract

*Colletotrichum higginsianum* is an ascomycete fungus causing anthracnose disease on numerous cultivated plants in the family *Brassicaceae*, as well as the model plant *Arabidopsis thaliana*. We report an assembly of the nuclear genome and gene annotation of this pathogen, which was obtained using a combination of PacBio long-read sequencing and optical mapping.

## GENOME ANNOUNCEMENT

*Colletotrichum* is a large genus of plant-pathogenic fungi that cause economically important anthracnose diseases on leaves and fruits of numerous monocot and dicot crops worldwide (1), although some species can grow as endophytes in symptomless plants (2). *C. higginsianum* attacks cultivated *Brassicaceae* and also *Arabidopsis thaliana*, providing a tractable model for analyzing fungal pathogenicity and plant responses (3). We previously sequenced the *C. higginsianum* genome using a combination of short reads from 454 FLX (350 bp) and Illumina (36 bp and 100 bp) platforms. This produced a highly fragmented assembly (GenBank accession number CACQ02000000) containing 10,269 contigs (*N*_50_ length = 6,150 bp) with a total length of 49.08 Mbp (4). In the resulting annotation of 16,172 protein-coding genes, many genes were truncated (~9%) or split between contigs (~7%), producing multiple gene calls. Here, we report a near-complete assembly of the nuclear genome of this fungus, which was obtained by combining long PacBio reads with the Optical Mapping System (5).

*C. higginsianum* strain IMI 349063 was originally isolated from leaves of *Brassica campestris* (3). High-molecular-weight genomic DNA was purified from mycelium using AXG100 columns (Macherey-Nagel) according to the manufacturer’s instructions. A size-selected library (~20 kb) was prepared and sequenced using the PacBio RSII sequencer and 15 single-molecule real-time (SMRT) cells with the P5-C3 polymerase-chemistry combination and 240-min movie time. The filtered sequence reads (7.06 Gbp, ~132.13× average coverage) were assembled *de novo* using HGAP version 3.0 and SMRT analysis version 2.3.0 software with default settings. Assembled sequences were aligned to the previously reported chromosome optical maps (4) for manual ordering and orientation of the contigs. Overlapping contigs were merged using Minimus2 (6). The assembled nuclear genome comprises 25 contigs (*N*_50_ contig length = 5.20 Mbp, maximum contig length = 6.04 Mbp), with a total length of 50.72 Mbp and a 51.86% G+C content. The 12 largest contigs (the predicted number of chromosomes) represent 99.3% of the assembly, and 11 chromosomes are completely sequenced from telomere to telomere. An additional 13 contigs, containing the rDNA repeats, were too small for alignment to the optical map. Based on consensus-calling results, the accuracy of the assembly is high (≥99.9%). Using the MAKER2 pipeline (7) to annotate the nuclear genome, a total of 14,651 protein-coding gene models were predicted.

By combining the long reads obtained from SMRT sequencing with optical mapping, we obtained a near-complete assembly of the nuclear genome of *C. higginsianum*, allowing a more accurate gene annotation that will facilitate future studies on the infection biology of this important model pathogen. The large contiguous genomic regions will be especially valuable for studying structural rearrangements, large secondary metabolism gene clusters, and the chromosome distribution of repetitive elements, pathogenicity-related genes, and epigenetic marks.

### Accession number(s).

This whole-genome shotgun project has been deposited at DDBJ/ENA/GenBank under the accession number LTAN00000000. The version described in this paper is version LTAN01000000.
